# Cardiac resynchronization therapy guided by late gadolinium-enhancement cardiovascular magnetic resonance

**DOI:** 10.1186/1532-429X-13-29

**Published:** 2011-06-13

**Authors:** Francisco Leyva, Paul WX Foley, Shajil Chalil, Karim Ratib, Russell EA Smith, Frits Prinzen, Angelo Auricchio

**Affiliations:** 1 Centre for Cardiovascular Sciences, Queen Elizabeth Hospital, University of Birmingham, UK; 2 University of Birmingham, Department of Cardiology, Good Hope Hospital, Sutton Coldfield, UK; 3 Departments of Physiology and Cardiothoracic Surgery, Cardiovascular Research Institute Maastricht, Maastricht, The Netherlands; 4 Fondazione Cardiocentro Ticino, Lugano, Switzerland

## Abstract

**Background:**

Myocardial scarring at the LV pacing site leads to incomplete resynchronization and a suboptimal symptomatic response to CRT. We sought to determine whether the use of late gadolinium cardiovascular magnetic resonance (LGE-CMR) to guide left ventricular (LV) lead deployment influences the long-term outcome of cardiac resynchronization therapy (CRT).

**Methods:**

559 patients with heart failure (age 70.4 ± 10.7 yrs [mean ± SD]) due to ischemic or non-ischemic cardiomyopathy underwent CRT. Implantations were either guided (+CMR) or not guided (-CMR) by LGE-CMR prior to implantation. Fluoroscopy and LGE-CMR were used to localize the LV lead tip and and myocardial scarring retrospectively. Clinical events were assessed in three groups: +CMR and pacing scar (+CMR+S); CMR and not pacing scar (+CMR-S), and; LV pacing not guided by CMR (-CMR).

**Results:**

Over a maximum follow-up of 9.1 yrs, +CMR+S had the highest risk of cardiovascular death (HR: 6.34), cardiovascular death or hospitalizations for heart failure (HR: 5.57) and death from any cause or hospitalizations for major adverse cardiovascular events (HR: 4.74) (all P < 0.0001), compared with +CMR-S. An intermediate risk of meeting these endpoints was observed for -CMR, with HRs of 1.51 (P = 0.0726), 1.61 (P = 0.0169) and 1.87 (p = 0.0005), respectively. The +CMR+S group had the highest risk of death from pump failure (HR: 5.40, p < 0.0001) and sudden cardiac death (HR: 4.40, p = 0.0218), in relation to the +CMR-S group.

**Conclusions:**

Compared with a conventional implantation approach, the use of LGE-CMR to guide LV lead deployment away from scarred myocardium results in a better clinical outcome after CRT. Pacing scarred myocardium was associated with the worst outcome, in terms of both pump failure and sudden cardiac death.

## Background

Cardiac resynchronization therapy (CRT) is an established treatment for symptomatic patients with heart failure, severe left ventricular (LV) systolic dysfunction and a prolonged QRS duration. The Cardiac Resynchronization Heart Failure (CARE-HF) study showed that CRT-pacing (CRT-P) was associated with 36% reduction in all-cause mortality. [1] The Comparison of Medical Therapy, Pacing and Defibrillation in Heart Failure (COMPANION) study showed that addition of a cardioverter defibrillator (CRT-D) leads to a greater survival benefit. [2] Additional benefits include reductions in heart failure hospitalizations as well as improvements in symptoms, exercise capacity and quality of life. [1-4]

The variability of the response to and outcome of CRT has been a subject of increasing attention [4,5]. Left ventricular (LV) lead position may be relevant in this respect. From the mechanical and electrophysiological perspectives, pacing scar is bound to be less effective than pacing viable myocardium. This notion is supported by cardiovascular magnetic resonance (CMR) [6,7] and nuclear scintigraphy [8] studies showing that myocardial scarring in the vicinity of the LV lead tip leads to a suboptimal response to CRT. These studies, however, have involved small numbers of patients, have not included patients with non-ischemic cardiomyopathy and have not addressed long-term clinical outcomes.

In this large study of consecutive patients undergoing CRT, we have assessed whether the use of late gadolinium enhancement (LGE)-CMR scan to guide deployment of the LV lead in a non-scarred segment of the LV free wall leads to a better long-term outcome from CRT than using a conventional implantation approach.

## Methods

### Patients

Inclusion criteria were as follows: heart failure in NYHA class III or IV; attendance to a dedicated heart failure clinic with the aim of achieving maximum tolerated treatment with angiotensin-converting enzyme inhibitors (ACE-I) or angiotensin II receptor blockers (ARBs), beta-blockers and spironolactone, and; a QRS duration ≥120 ms; LVEF ≤ 35%. Exclusion criteria were: contraindications to cardiac pacing; myocardial infarction or acute coronary syndrome within the previous month; severe structural valvular heart disease; presence of comorbidities likely to threaten survival for 12 months. All participants had undergone coronary angiography. The diagnosis of heart failure was made on the basis of echocardiographic evidence of LV systolic dysfunction. The diagnosis of ischemic cardiomyopathy was made if LV systolic dysfunction was associated with a history of myocardial infarction [9] and if there was angiographically documented coronary heart disease (> 50% stenosis in ≥ 1 coronary arteries). The findings of LGE-CMR were also used to ascertain the etiology of heart failure: [10] LV dysfunction in combination with transmural or subendocardial LGE was regarded as ischemic cardiomyopathy whereas LV dysfunction and no LGE, patchy uptake or mid-wall LGE was regarded as non-ischemic cardiomyopathy. The study conforms with the Declaration of Helsinki. This study was approved by the local Ethics Committee.

### Study design

This study consisted of patients who underwent CRT on the basis of accepted indications in the period from September 2000 to June 2009. As national guidance and funding for CRT-D in the United Kingdom was not issued until 2007, [11] CRT-P was only CRT modality available for most patients.

Patients underwent a clinical assessment on the day prior to implantation and at 1, 3, and every 6 months following device implantation. The LGE-CMR scan was undertaken within a month prior to implantation. In patients who died, the clinical and echocardiographic data at follow-up pertains to the latest available follow-up.

Prior to the demonstration that myocardial scarring at the site of LV lead deployment is associated with a suboptimal response to CRT, [7] the implanters were blinded to the results of the LGE-CMR. Following this demonstration, the LGE-CMR was used to guide LV lead deployment. For analysis, patients were grouped into the following categories: +CMR and pacing scar (CMR+S, n = 43); CMR and not pacing scar (+CMR-S, n = 166), and; LV pacing not guided by CMR (-CMR, n = 350).

### Clinical assessment and echocardiography

This included evaluation of NYHA functional class and a 6-min hall walk test [12]. Response in terms of the composite clinical score (CCS) was defined as: survival for one year following implantation; no hospitalizations for heart failure for one year following implantation, and; improvement by ≥1 NYHA classes or by ≥25% in 6-min walking distance. Two-dimensional echocardiography was performed using a Vivid Systems 5 and 7 scanners (General Electric Healthcare Worldwide, Slough, United Kingdom). An echocardiographic response, denoting LV reverse remodeling, was defined as a ≥15% reduction in LV end-systolic volume (LVESV) at follow-up.

### CMR

Images were acquired on a 1.5 Tesla scanner (Signa, General Electric Healthcare Worldwide, Slough, United Kingdom) using a phased array cardiac coil during repeated 8-second breathholds. A short axis stack of left ventricular images was acquired using a steady state in free precession (SSFP) sequence (repetition time 3.0 to 3.8 ms; excitation time 1.0 ms; image matrix 224 × 224; field of view 36-42 cm; flip angle 45°) in sequential 8 mm slices (2 mm interslice gap) from the atrioventricular ring to apex. For the LGE-CMR study, gadolinium-diethylenetriamine pentaacetic acid (0.1 mmol/kg) was administered intravenously and images were acquired after 10 minutes using a segmented inversion-recovery technique in identical short-axis slices, as previously described [7]. Inversion times were adjusted to null normal myocardium (260 to 400 ms). Infarct volume was calculated in cm^3 ^by multiplying the planimetered area in each segment by the slice thickness. Scar volume was expressed as a % of LV myocardial volume in the diastolic phase.

### Device therapy

Patients underwent transvenous CRT device implantations using cephalic, subclavian or femoral vein approaches. If a LGE-CMR was available, implanters were asked to deploy the LV lead away from scarred myocardium, if at all possible. The operator, however, was given freedom to deploy the LV lead elsewhere if other lead parameters or pacing characteristics were unsatisfactory.

### Lead position

**Figure 1:**
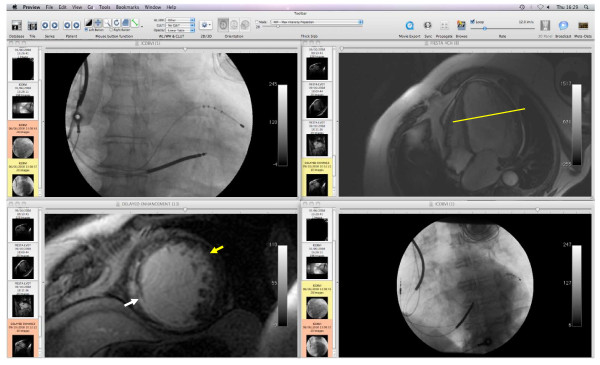
**Mapping LV lead positions**. Example of the main screen of the software programme used for mapping LV lead positions. The longitudinal distance from the atrioventricular plane to the lead tip, in a base-to-apex direction, is quantified in mm using the 30° right anterior oblique fluoroscopic view (upper left hand panel). This longitudinal distance is transposed to the four-chamber CMR view (upper right hand panel), so as to determine the LGE-CMR short axis slice (yellow line, left lower panel) that corresponds to the LV lead tip position. The 30° left anterior oblique fluoroscopic view (right lower panel) is then used to determine the circumferential position (yellow arrow). The longitudinal and circumferential coordinates permit localization of the LV lead tip in relation to myocardial segments [35] and myocardial scars, which appear as white enhancement on LGE-CMR (white arrow).

The segmental position of the LV lead tip was determined with reference to its longitudinal (base-to-apex) and its circumferential position, using a dedicated imaging programme (available free at http://www.osirix.com) (Figure 1). The LV lead tip was considered to be in a scarred segment if this contained scar in any distribution (transmural or non-transmural). Lead positions were assessed retrospectively by a senior radiographer and by a cardiologist who were blinded to the clinical outcome data.

### Follow-up

Patients were entered into the study after optimization of medical therapy and a successful implantation. Thereon, they were followed-up in a dedicated CRT clinic. Patients in sinus rhythm underwent transmitral Doppler-directed optimization of atrioventricular delay [13] prior to discharge and at every scheduled visit thereafter. Backup atrial pacing was set at 60 beats/min, and the pacing mode was set to DDD with an interventricular delay of 0-4 ms, depending on the manufacturer. For patients in chronic atrial fibrillation, right ventricular and LV leads were implanted and a CRT generator was used, plugging the atrial port and programming the generator to a ventricular-triggered mode.

### Endpoints

The clinical endpoints considered were cardiovascular mortality; the composite of death from any cause or an unplanned hospitalization for major adverse cardiovascular events (MACE), which included cardiac transplantation, hospitalizations for worsening heart failure, myocardial infarction, acute coronary syndromes, arrhythmia, stroke or pulmonary embolism; the composite end point of death from any cause and unplanned hospitalization with worsening heart failure. For all endpoints, the first event was included in analyses. Sudden cardiac death was defined as a "natural, unexpected death due to cardiac causes, heralded by an abrupt loss of consciousness within one hour of the onset of acute symptoms."[14]Death from pump failure was defined as 'death after a period of clinical deterioration in signs and symptoms of heart failure despite medical treatment'.[15] Clinical outcome data was collected prospectively through medical records, and where appropriate, from interviews with patient's caregivers. Information regarding clinical outcome was collected by an investigator who was blinded to the results of the CMR study and LV lead position data.

### Statistical analysis

Continuous variables are expressed as mean ± standard deviation (SD). Normality was tested using the Shapiro-Wilk test (the W-statistic). Comparisons between normally distributed continuous variables were made using ANOVA with Fisher's Protected Least Significance Difference test for multiple comparisons. Categorical variables were analyzed using chi-squared tests. Group differences with respect to the various endpoints was explored using Kaplan-Meier survival curves and the log-rank test (Mantel-Cox). Univariate and multivariable Cox proportional hazards analyses were also used to explore the relationships between the groups and the various endpoints. Statistical analyses were performed using Statview (Cary, NC) and SPSS 13.0 (Chicago, Illinois). A two-tailed *p *value of < 0.05 was considered statistically significant.

## Results

In the period September 2000 to June 2009, 559 consecutive, successful CRT implantations were undertaken for standard indications at a single centre (Good Hope Hospital). A total of 87/559 (16%) underwent CRT-D whilst the remainder underwent CRT-P. In the whole cohort, 367/559 (66.7%) patients had ischemic cardiomyopathy and 192/559 (34.3%) had non-ischemic cardiomyopathy.

As shown in Table 1, there were significant group differences with respect to pre-implant age, NYHA class, QRS duration, history of coronary artery bypass operation and presence of chronic atrial fibrillation. Over a maximum follow-up period of 3323 days (median: 666 days), there were 181 deaths from all causes. Of these, 149 were due to cardiovascular causes, including 2 patients who underwent cardiac transplantation.

**Table 1:** Clinical Characteristics of the Study Group

					P		
	All	A -CMR	B +CMR-S	C +CMR+S	B vs A	C vs A	C vs B
*N*	559	350	166	43			
Age, yrs	70.4 ± 10.7	71.6 ± 10.7	68.3 ± 10.6	68.5 ± 10.6	0.0007	0.0704	0.8714
Men, n (%)	436 (78)	275 (79)	123 (74)	38 (88)	0.1203		
NYHA class	3.31 ± 0.5	3.35 ± 0.5	3.20 ± 0.40	3.37 ± 0.49	0.0004	0.8115	0.0287
III	385 (69)	225 (64)	133 (80)	27 (63)			
CRT-D, n (%)	87 (16)	52 (15)	27 (16)	8 (19)	0.7799		
**Co-morbidity**, No. (%)							
Diabetes mellitus	90 (16)	55 (16)	25 (15)	10 (24)	0.4248		
Hypertension	149 (27)	93 (17)	44 (27)	12 (28)	0.9575		
Coronary artery bypass	112 (20)	65 (19)	30 (18)	17 (40)	0.0048		
**Medication**, No. (%)							
Loop diuretics	495 (89)	314 (90)	144 (87)	37 (86)	0.2619		
ACE-I or ARB	498 (89)	306 (87)	151 (91)	41 (95)	0.3392		
Beta-blockers	300 (54)	177 (51)	97 (58)	26 (60)	0.2641		
Spironolactone	224 (40)	138 (39)	65 (39)	17 (40)	0.9142		
**ECG variables**							
Chronic atrial fibrillation, No. (%)	119 (21)	86 (25)	29 (17)	4 (9)	0.0249		
QRS duration, ms	154.3 ± 28.5	158.5 ± 29.5	148.6 ± 25.4	144.5 ± 26.0	0.0003	0.0022	0.3860
**Echocardiography**							
LVEDV, mL	259.0 ± 109.0	252.5 ± 107.2	268.3 ± 115.1	267.5 ± 97.2	0.1938	0.4489	0.9703
LVESV, mL	200.9 ± 94.5	196.5 ± 92.2	207.2 ± 101.2	206.0 ± 83.8	0.3109	0.5820	0.9466
LVEF, %	23.5 ± 10.1	23.2 ± 10.4	24.1 ± 9.9	23.7 ± 8.6	0.4346	0.7832	0.8465

+CMR-S = CMR showing no scar at the LV pacing position; +CMR+S = CMR showing scar at the LV pacing position; -CMR = non-CMR guided group.

### Clinical endpoints

**Figure 2:**
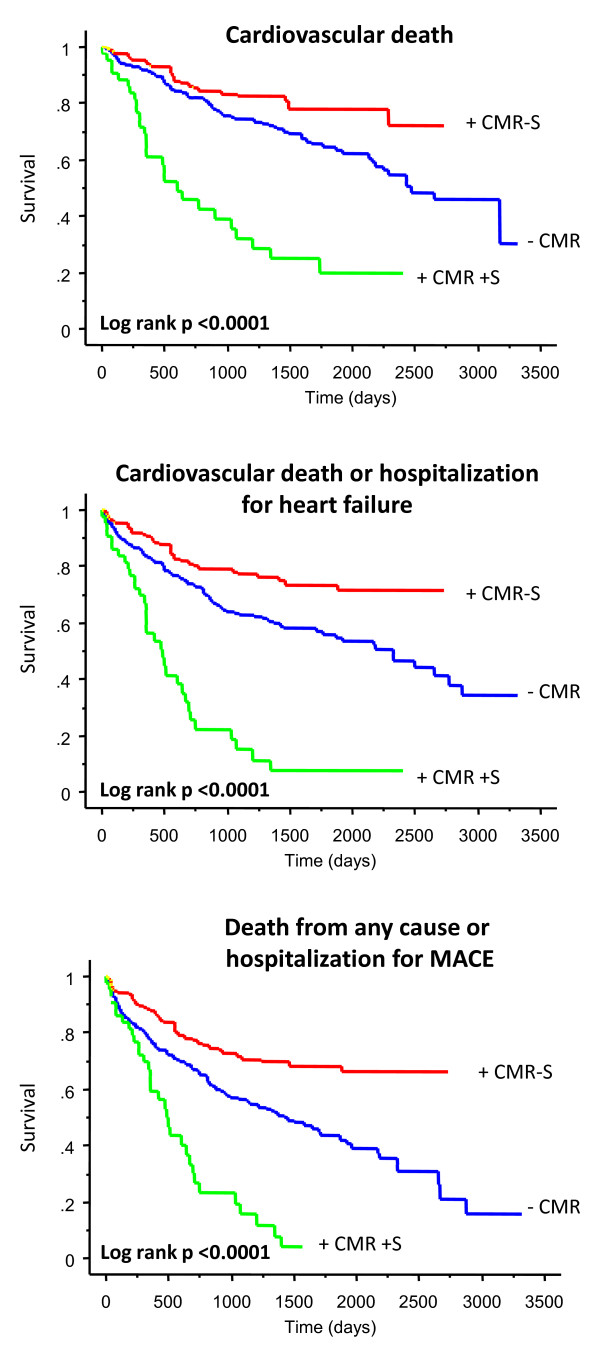
**Clinical outcome of CRT according to implantation strategy**. +CMR-S = group with CMR showing no scar at the LV pacing position; +CMR+S = group with CMR showing scar at the LV pacing position; -CMR = non-CMR guided group.

As shown in Figure 2, the +CMR+ S group had the highest risk of cardiovascular death, the composite endpoint of cardiovascular death or hospitalizations for heart failure and the composite endpoint of death from any cause or hospitalizations for MACE (all P < 0.00001). In Cox proportional hazards models, age, NYHA class, QRS duration, history of CABG and of chronic AF were entered as covariables. With reference to the adjusted HRs (Table 2), the +CMR+S group had the highest risk of cardiovascular death (HR: 6.34), the composite endpoint of cardiovascular death or hospitalizations for heart failure (HR: 5.57) and the composite endpoint of death from any cause or hospitalizations for MACE (HR: 4.74) (all P < 0.0001), compared with the +CMR-S group. The -CMR group had a intermediate risk of meeting these endpoints, with HRs of 1.51 (P = 0.0726), 1.61 (P = 0.0169) and 1.87 (p = 0.0005), respectively.

**Table 2:** Effects of implantation strategy on clinical outcomes

	Cardiovascular death		Cardiovascular death / hospitalizations for HF		Death from any cause / hospitalizations for MACE	
	HR (95% CI) *	p	HR (95% CI) *	p	HR (95% CI) *	p
+CMR+S	6.34 (3.64 to 11.0)	< 0.0001	5.57 (3.40 to 9.14)	< 0.0001	4.74 (2.95 to 7.62)	< 0.0001
-CMR	1.51 (0.96 to 2.36)	0.0726	1.61 (1.09 to 2.38)	0.0169	1.87 (1.31 to 2.66)	0.0005

*, Hazard ratios (HR) and 95% confidence intervals (CI) refer to the risk of meeting the endpoints in comparison to the group in which CMR was performed and the LV lead was deployed in non-scarred myocardium. Adjustment has been made for age, NYHA class, QRS duration, history of CABG and of chronic atrial fibrillation in Cox proportional hazards analyses. +CMR+S = CMR showing scar at the LV pacing position; -CMR = non-CMR guided group.

In further analyses, the +CMR-S group was split into patients with non-ischemic cardiomyopathy (n = 74) and patients with ischemic cardiomyopathy (n = 92). In Cox proportional hazards analyses, there were no differences with respect to cardiovascular death (HR: 0.59 [95% CI: 0.26 to 1.31], p = 0.1906) or any other endpoint (data not shown). The +CMR-S group was also subdivided according to scar burden. In receiver operator characteristic curves (ROC), a scar burden cut-off of ≥10% was associated with a sensitivity of 81.1% and a specificity of 40.0% for the detection of cardiovascular death (p < 0.0001). A scar burden of < 10% predicted survival from cardiovascular death (HR: 0.37, 95% confidence interval [CI]: 0.17 to 0.81, χ^2 ^= 6.12, p = 0.0134), the composite endpoint of cardiovascular death or hospitalizations for heart failure (HR: 0.36, 95% CI: 0.17 to 0.75, χ^2 ^= 7.60, p = 0.0058) and the composite endpoint of death from any cause or hospitalizations for MACE (HR: 0.50, 95% CI: 0.26 to 0.98, χ^2 ^= 4.09, p = 0.0432).

### Mode of death

Out of 149 cardiovascular deaths, 107 were due to pump failure (including 2 patients who underwent cardiac transplantation), 39 were sudden and 3 were due to myocardial infarction. As shown in Table3, +CMR+S group had the highest risk of death from pump failure (HR: 5.40, p < 0.0001) and sudden cardiac death (HR: 4.40, p = 0.0218). As evidenced by the much higher **χ^2^**, the association between +CMR+S and pump failure ( **χ^2 ^**= 29.1) was stronger than with sudden cardiac death (**χ^2 ^**= 5.26).In the +CMR-S group, patients with a scar burden of < 10% had a lower risk of death from pump failure (HR: 0.28, 95% CI: 0.11 to 0.71, χ^2 ^= 7.34, p = 0.0067), but not of sudden cardiac death (HR: 0.43, 95% CI: 0.07 to 2.59, χ^2 ^= 0.85, p = 0.3581), compared with patients with a scar burden of ≥10%.

**Table 3:** Effects of implantation strategy on mode of death

	Death from pump failure			Sudden cardiac death		
	HR (95% CI) *	χ^2^	p	HR (95% CI) *	χ^2^	p
+CMR+S	5.40 (2.92 to 9.94)	29.1	< 0.0001	4.40 (1.24 to 15.62)	5.26	0.0218
-CMR	1.12 (0.67 to 1.85)	0.13	0.6847	2.93 (1.11 to 7.76)	4.71	0.0299

*, Hazard ratios (HR) and 95% confidence intervals (CI) refer to the risk of meeting the endpoints in comparison to the group in which CMR was performed and the LV lead was deployed in non-scarred myocardium. Adjustment has been made for age, NYHA class, QRS duration, history of CABG and of chronic atrial fibrillation in Cox proportional hazards analyses. +CMR+S = CMR showing scar at the LV pacing position; -CMR = non-CMR guided group.

### Clinical response

**Figure 3:**
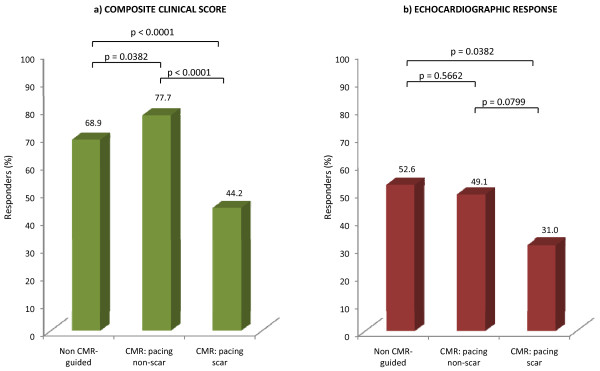
**Symptomatic and echocardiographic response according to implantation strategy**. Numbers refer to the proportion of patients meeting the respective endpoints by the end of the study.

NYHA class was available in 532 patients at a median follow-up period of 287 days (interquartile range: 463.7 days). Of these, 428 (80%) had improved by ≥1 class at follow-up. Baseline 6-min walking distance was available in 333 patients (217.8 ± 113.8 m). Follow-up 6-min walk tests were available in 226 patients. In the whole cohort, 6-min walking distance increased by ≥25% in 106 (51.2%) patients. Of the 553 patients in whom there was sufficient data to classify in terms of the composite clinical score, 385 (70%) were classified as responders. As shown in Figure 3a, the CMR-S group had the best clinical response, compared with the CMR+S group (P < 0.00001) and the -CMR group (P = 0.0382)

In the +CMR-S group, a scar burden of < 10% was not associated with a better composite clinical score compared with a scar burden of ≥10% (p = 0.7281).

### LV reverse remodeling

Amongst patients with complete follow-up echocardiography at the last available clinical visit (n = 320), LV reverse remodeling was less pronounced in the +CMR+S group (31%) than in the -CMR group (p = 0.0382). There was a trend towards a better response in the CMR-S group than in the +CMR+S group, but this was not statistically significant (P = 0.0799) (Figure 3b).

In the +CMR-S group a scar burden of < 10% was not associated with a better LV reverse remodeling response compared with a scar burden of ≥10% (p = 0.3500).

## Discussion

The hypothesis addressed in this study is that, compared with a conventional approach, the use of LGE-CMR for guiding LV lead deployment away from scarred myocardium leads to a better outcome from CRT. We have shown that scarring in the myocardial segment subtended by the LV lead tip has a dramatic negative effect outcome of and response to CRT. Compared with pacing non-scar, pacing scar was associated with an over 6-fold increase in the risk of cardiovascular death. An implantation strategy which did not include LGE-CMR was associated with a worse outcome than a strategy of pacing non-scar, in terms of the composite end points and the composite clinical score. Although pacing scar had a particularly strong relationship with death from pump failure, it was also associated with a higher risk of sudden, presumed arrhythmic, death.

The importance of myocardial viability in determining the response to treatments, such as revascularization [16-18] and beta-blockade for heart failure, [19] is well recognized. The concept that myocardial viability of the myocardium subtended by the LV lead is important in determining the response to CRT has been supported by several small studies [6-8]. In the present study of a large cohort of patients with ischemic or non-ischemic cardiomyopathy, we have found that, compared to LV deployment over non-scarred myocardium, deployment over scarred myocardium is associated with a higher risk of cardiovascular death, hospitalizations for heart failure and for MACE. In addition, pacing scarred myocardium was also associated with a markedly reduced clinical response in terms of the composite clinical score. These findings are consistent with the fact that pacing a scar is associated increased duration [20,21] and fragmentation of the QRS complex, as well as suboptimal mechanical resynchronization [22]. Moreover, it has been shown that myocardial scars are not readily excitable [23] and that they reduce the volume of excitable myocardium available to a LV pacing stimulus [24].

We have observed a trend towards a less pronounced LV reverse remodeling in the group of patients with LV leads deployed over scarred myocadium. Although the difference between the +CMR+S and the -CMR groups were statististically significant, with a lower response in the +CMR+S group, the difference between the +CMR+S and the +CMR-S groups was not significant. This, however, is likely to be due to the relatively low numbers of patients included in these subgroups. Notwithstanding, the trend towards less LV reverse remodeling in the +CMR+S group is consistent with other studies [22] and could partly explain the associated effects on outcome and response. Importantly, however, LV remodeling is not the only mechanism involved in CRT. Relief of the diastolic ventricular interaction, [25] and a reduction in mitral regurgitation [26] are amongst the additional mechanisms that are at play in CRT. These mechanisms are not necessarily dependent on reverse LV remodeling [27,28].

Compared with the +CMR-S group, the +CMR+S group also had a higher risk of sudden cardiac death. These findings have emerged in the context that pacing over scar or the border zone of scar can be arrhythmogenic.[29-32]In a study of 47 ICD candidates, Schmidt et al a relationship between inducibility of arrhythmias and heterogenetity in the border zone of scar.[33]In addition, the extent of the border zone of scar is a strong predictor of spontaneous ventricular arrhythmias and subsequent ICD therapy in patients with ischemic cardiomyopathy, with or without a history of ventricular arrhythmias.[34]

In analyses of patients in whom the LV lead had been deployed away from scarred myocardium, a low scar burden (< 10%) was associated with a better clinical outcome, in terms of all endpoints. In terms of mode of death, a scar burden of > 10% was associated with a higher risk of death from pump failure, but not sudden cardiac death. These findings are perhaps not surprising, as increasing scar burden equates with poor myocardial function. Importantly, however, scar burden did not appear to influence LV reverse remodelling, nor the composite clinical score. On the basis of these findings, a high scar burden (≥10% ) in +CMR-S patients does not necessarily discount a symptomatic benefit from CRT.

Several studies have shown that an ischemic etiology has a negative effect on the outcome of CRT. In our whole cohort, etiology of heart failure did indeed emerge as a predictor of the various endpoints. However, this was no longer the case when patients in the +CMR+S group were excluded. This indicates that the observed differences in the response to and outcome of CRT is due to deployment of the LV lead over scarred myocardium. Admittedly, the numbers included in this subanalysis are relatively small and one cannot exclude the possibility that the outcome of CRT is better in non-ischemic cardiomyopathy, regardless of LV lead position. Notwithstanding, it appears that the outcome of CRT in ischemic cardiomyopathy approximates to that of non- ischemic cardiomyopathy, as long as the LV lead is deployed over non-scarred myocardium.

## Limitations

This is an observational study and therefore, our findings should be interpreted with caution. The groups analyzed were not matched for baseline variables and although this was taken into account in statistical analyses, a biological effect of the covariables cannot be discounted. These findings should ideally be explored in a randomized, controlled study. Given the currently available evidence on the detrimental effects of myocardial scarring at the LV pacing site, however, it is unlikely that a randomized study would be undertaken. We have not included a control group not treated with CRT and therefore, we cannot determine whether patients in highest risk category (+CMR+S) benefit to the same degree as patients treated with drug treatment alone. A randomized study of drug treatment versus CRT, however, is no longer ethically justifiable. This study did not address the relationship of LV lead pacing site to regional mechanical dyssynchrony, which is thought to be valuable in determining response.

## Conclusions

We conclude that, compared with a conventional implantation approach, the use of LGE-CMR to guide LV lead deployment away from scarred myocardium results in a better clinical outcome after CRT. On this basis, we would recommend the use of LGE-CMR in the routine pre-implant work-up of patients undergoing CRT.

## Conflict of interest

The authors declare that they have no competing interests.

## Authors' contributions

FL conceived the hypothesis, contributed to clinical procedures, data collection, data analysis and writing of the manuscript. PWXF contributed to clinical procedures, data collection, data analysis and writing of the manuscript. SC contributed to clinical procedures and data collection. REAS contributed to clinical procedures and revised the manuscript. FP and AA contributed to study design and writing of the manuscript. All authors read and approved the final manuscript.
